# The Zinc-Finger Thylakoid-Membrane Protein FIP Is Involved With Abiotic Stress Response in *Arabidopsis thaliana*

**DOI:** 10.3389/fpls.2018.00504

**Published:** 2018-04-18

**Authors:** Karina L. Lopes, Ricardo A. O. Rodrigues, Marcos C. Silva, Wiliane G. S. Braga, Marcio C. Silva-Filho

**Affiliations:** Departamento de Genética, Escola Superior de Agricultura Luiz de Queiroz, Universidade de São Paulo, Piracicaba, Brazil

**Keywords:** *Arabidopsis*, zinc-finger, stress tolerance, gene regulation, FtsH

## Abstract

Many plant genes have their expression modulated by stress conditions. Here, we used *Arabidopsis* FtsH5 protease, which expression is regulated by light stress, as bait in a yeast two-hybrid screen to search for new proteins involved in the stress response. As a result, we found FIP (FtsH5 Interacting Protein), which possesses an amino proximal cleavable transit peptide, a hydrophobic membrane-anchoring region, and a carboxyl proximal C_4_-type zinc-finger domain. *In vivo* experiments using FIP fused to green fluorescent protein (GFP) showed a plastid localization. This finding was corroborated by chloroplast import assays that showed FIP inserted in the thylakoid membrane. *FIP* expression was down-regulated in plants exposed to high light intensity, oxidative, salt, and osmotic stresses, whereas mutant plants expressing low levels of *FIP* were more tolerant to these abiotic stresses. Our data shows a new thylakoid-membrane protein involved with abiotic stress response in *Arabidopsis thaliana*.

## Introduction

Plants are constantly exposed to biotic and abiotic environmental stress conditions. These parameters include water and nutrients availability, microorganism population in soil, predators, pests, salinity, temperature variance, light incidence, etc. As a mechanism of defense to environmental parameters variations, plants are subjected to molecular, physiological and/or phenotypic changes. Gene expression modulation is one of the most common responses to stressful conditions (Shinozaki and Yamaguchi-Shinozaki, 2000; Chen et al., 2002; Kreps et al., 2002; Seki et al., 2002; Yamaguchi-Shinozaki and Shinozaki, 2006; Fujita et al., 2009).

In *Arabidopsis*, the thylakoid FtsH complex is directly involved in stress response (Kamata et al., 2005; Khatoon et al., 2009; Kirchhoff et al., 2011; Yoshioka and Yamamoto, 2011; Bove et al., 2012; Herbstova et al., 2012), in particular the FtsH5 protease, which is involved with light stress response (Sakamoto et al., 2002). FtsH proteases belong to the AAA+ (ATPase Associated with diverse cellular Activities) family (Tomoyasu et al., 1993; Ogura and Wilkinson, 2001; Kato and Sakamoto, 2010; Liu et al., 2010; Nixon et al., 2010) and their functions in cells are known to some extent (Ogura et al., 1999; Adam et al., 2006). FtsH protein was first described in *Escherichia coli*, where it is involved in the proteolysis of membrane proteins (Ito and Akiyama, 2005; Wagner et al., 2012) and, most importantly, the degradation of heat shock sigma factor σ^32^ (Tomoyasu et al., 1995; Shotland et al., 1997; Ogura et al., 1999). In fact, the name FtsH is derived from Filamentation Temperature Sensitive H due to the phenotype of the *E. coli* mutant Y16 lacking the *FtsH* gene, which is unable to degrade sigma factor σ^32^ (Santos and De Almeida, 1975; Ogura et al., 1991; Begg et al., 1992; Tomoyasu et al., 1995). While bacteria have only one copy of the *FtsH* gene (Ogura et al., 1991; Akiyama et al., 1995; Bieniossek et al., 2006), 12 FtsH orthologues have been found in *Arabidopsis thaliana*, and nine of these are targeted to chloroplasts (Takechi et al., 2000; Sakamoto et al., 2002, 2003; Sokolenko et al., 2002).

Light stress response mechanism is mediated by thylakoid FtsH proteases by the degradation of photodamaged PSII D1 protein with the support of Deg proteases (Lindahl et al., 2000; Bailey et al., 2002; Kato et al., 2009, 2012; Kato and Sakamoto, 2010, 2014). The FtsH complex also participates in thylakoid membrane formation and the degradation of unassembled proteins (Ostersetzer and Adam, 1997; Lindahl et al., 2000; Zaltsman et al., 2005; Jarvi et al., 2016). FtsH proteases require ATP hydrolysis and zinc ions to activate proteolysis (Herman et al., 2003; Ito and Akiyama, 2005; Bieniossek et al., 2009; Wagner et al., 2012). In higher plants, light stress promotes conformational changes in FtsH protease monomers, leading to formation of the heterohexameric complex formed by type A (FtsH1 and FtsH5) and type B (FtsH2 and FtsH8) in a 2:4 ratio (Yu et al., 2004; Yoshioka and Yamamoto, 2011). Light stress also induces grana relaxation, facilitating the traffic of FtsH complexes through unstacked thylakoid membranes and access to damaged PSII D1 proteins (Khatoon et al., 2009; Kirchhoff et al., 2011; Herbstova et al., 2012).

Based on the importance of FtsH proteases in the light stress response, and considering that type B FtsH proteases activity are important for proper chloroplast development (Zhang et al., 2010), a type A protease FtsH5, was used as bait in a yeast two-hybrid screen to search for new proteins involved in the stress response. A new protein (At5g02160) was found interacting with FtsH5 and has been named FIP (FtsH5 Interacting Protein).

FIP possess a zinc-finger domain (type C_4_) with two CXXCXGXG conserved repeats. A zinc-finger domain is characterized by the presence of two CXXCXGXG motifs (where X is any amino acid), which is present in DNAJ proteins (Shi et al., 2005). The zinc-finger domain, as observed in DNAJ proteins, is repeated twice. The first is responsible for the chaperone activity of DNAJ, while the second acts mainly in the interaction with the partner DNAK (Tang and Wang, 2001). The 40-kDa DNAJ provides activity regulation, mainly through binding of the J domain to the ATPase region of DNAK (Walsh et al., 2004). In the absence of a conserved J domain, as observed in FIP and other DNAJ-like proteins, it is verified that those proteins usually demonstrate chaperone activity non-dependent of Hsp70, by substrate interaction and stabilization.

In the present study, we show that a transmembrane protein containing a zinc-finger domain interacts with FtsH5 in a yeast two-hybrid assay, confirmed by pull-down experiments. FIP colocalizes with the FtsH5 in thylakoids, and is related to abiotic stress response, since *fip* knockdown mutants are more tolerant to abiotic stresses and that *FIP* expression is down-regulated in response to abiotic stress. Our data describes a new thylakoid targeted protein that is directly involved in the abiotic stress response.

## Materials and Methods

### *In Silico* Analysis

*Arabidopsis FIP* and *FtsH* sequences were obtained from TAIR^1^. Transmembrane domains were predicted by TMPred^2^.

Data from the AtGenExpress Project^3^ (Kilian et al., 2007) is part of the TAIR database (The *Arabidopsis* Information Resource, see foot note text 1) was analyzed with the package R/Bioconductor (Gentleman et al., 2004) and normalized by robust multi-array average [RMA; (Irizarry et al., 2003)].

*FIP* homologous and *FtsH* proteases type A sequences were identified through blastn algorithm, by means of reciprocal blast. The sequences were compared to the non-redundant (nr/nt) GenBank database^4^ and the Phytozome database^5^ and selected based on an adequate *e*-value threshold.

### Yeast Two-Hybrid Assays

The AH109 strain of *Saccharomyces cerevisiae* (MATa trp1-901 leu2-3, 112 ura3-52 his3-200 gal4 gal80 LYS2::GAL1-HIS3 GAL2-ADE2 met2::GAL7-lacZ) was used in all experiments. For the screening using an *Arabidopsis* library, the yeast strain containing the full-length *FtsH5* as bait was employed. The LiAc/SS carrier DNA/PEG transformation method was used (Gietz and Schiestl, 2007). Plates were maintained for 14 days at 30°C. Positive colonies were rescreened on SC-leu-trp-his and SC-leu-trp-ade medium plates and incubated at 30°C for 48 h. Clone growth in both plates was considered positive and subjected to DNA sequencing.

### Protein Expression in *E. coli* and Protein Purification

An isolated colony of transformed *E. coli* strain BL21 was inoculated into LB medium containing the appropriate antibiotic and grown at 37°C and 200 rpm for 18 h. The preculture was inoculated into LB medium containing the appropriate antibiotics at a ratio of 1:100 of the total culture volume and incubated with agitation at 37°C until the optical density reached 0.6. Protein expression was induced with IPTG at a final concentration of 1 mM. The culture was incubated at 30°C for 4 h. The cells were centrifuged, and the pellet was frozen at -70°C.

FtsH5 was purified using a nickel resin Ni-NTA Spin Columns kit from QIAGEN according to the manufacturer’s instructions. For purification of the glutathione S-transferase (GST) GST-FIP fusion, Glutathione Superflow resin from QIAGEN was used according to the manufacturer’s instructions.

### GST Pull-Down and Western Blotting

To each tube was added 50 ml of agarose beads and 1 μg purified FtsH5. To the first tube only was added incubation buffer (50 mM Tris–HCl, 200 mM NaCl, 1 mM EDTA, 1% Nonidet P-40, 1 mM DTT, 10 mM MgCl 2, pH 8.0). To the second tube was added 25 mg of the purified GST protein and incubation buffer. To the third tube was added 25 mg of the GST-FIP fusion and incubation buffer. All volumes were filled to 500 ml, followed by incubation for 4 h on ice with gentle agitation. The beads were then washed 5 times with 1 ml of incubation buffer and resuspended in 50 μl of SDS–PAGE sample buffer.

After SDS–PAGE, the samples were transferred to nitrocellulose membrane (Bio-Rad 0.45 μm). The membrane was then incubated in TBS-T buffer with 0.2% BSA and an adequate amount of primary antibody for 2 h. The membrane was then washed three times for 15 min with TBS-T buffer and used for incubation with an adequate amount of secondary antibody in TBS-T buffer with 0.2% BSA for 1 h. The membrane was then washed three times for 15 min with TBS-T buffer and used for film exposure after addition of 1 ml of substrate (Bio-Rad) for alkaline phosphatase.

### Transient Expression and Confocal Microscopy Analysis

Leaves of *N. tabacum* cv. SR1 were used for *Agrobacterium*-mediated transient transformation. A single *Agrobacterium* colony was incubated in five ml of LB medium containing appropriated antibiotics, 50 μM acetosyringone, and 10 mM MES (pH 5.6). The culture was incubated at 28°C for 16 h and 1.5 ml was centrifuged at 13,000 rpm for 1 min. One ml of 10 mM MgCl_2_ was used to resuspend the pellet and the OD600 of the culture was adjusted to 0.2 and 100 μM of acetosyringone. *N. tabacum* leaves were infiltrated using a syringe without a needle. Mesophyll protoplasts were prepared as described by Carneiro et al. (1993). After 4 and 5 days of infiltration, the microscope analysis was conducted using an Olympus FV1000 confocal microscope. Excitation filters were, respectively, for GFP and chlorophyll autofluorescence: 488 and 635 nm; Emission filters were, respectively, for GFP and chlorophyll autofluorescence: 510–550 nm and 670–700 nm. Images were obtained with the following software: Olympus FluoView FV10-ASW.

### Chloroplast Import and Thylakoid Integration

SP6 polymerase transcription kit (Promega) was used to produce radiolabeled precursors. The RNA was translated with a wheat germ kit (homemade) with ^3^[H]leucine (Cline, 1986). 60 mM leucine in 2× IB (IB, import buffer; 1× = 50 mM HEPES/KOH, pH 8.0, 0.33 M sorbitol) was used to dilute the products of translation, prior to use.

For chloroplasts isolation, nine to ten-day-old *Pisum sativum* cv. Laxton’s Progress 9 seedlings were used according to Cline (1986) and chlorophyll was determined as described (Arnon, 1949). Intact chloroplasts were used to produce lysates, which was the base to produce thylakoids and stroma (Cline et al., 1993). Radiolabeled precursors importation into intact chloroplasts, chloroplast lysates, or thylakoids (0.33 mg chlorophyll/ml or equivalent) was conducted at 25°C, 5 mM MgATP, and 70–100 μE m^-2^sec^-1^ white light (Cline et al., 1993). Lysis of recovered chloroplasts occurred by adding 20 mM HEPES/KOH, pH 8.0 on ice for 5 min. Centrifugation for 8 min at 3200 g was employed to separate thylakoids from stroma. IB was used to wash and 100 mM NaOH to extract the thylakoid membrane. Thermolysin treatment occurred according to Summer et al. (2000), using 1 μg thermolysin per μg chlorophyll for 40 min at 4°C and the thermolysin was inactivated with IB with 14 mM EDTA. SDS–PAGE and fluorography was used to analyze the samples.

### Multiple Sequence Alignment and Phylogenetic Inference

Multiple sequence alignments (MSAs) were generated with the TranslatorX server^6^ (Abascal et al., 2010) using MAFFT v.7 software (Katoh and Standley, 2013) and curated in Jalview v.2.8.0 or Gblocks [standard parameters; (Castresana, 2000; Talavera and Castresana, 2007)] for large amount of data. MSAs were visualized in Jalview v.2.8.0 (Waterhouse et al., 2009) and trimmed for short, redundant, largely incomplete, and poorly aligned sequences.

Bayesian phylogenetic inference was conducted using MrBayes v.3.2.1 (Ronquist and Huelsenbeck, 2003) for the tree topology and branch length based on MSAs and substitution models. For this procedure, 2 × 10^6^ MCMC (Markov chain Monte Carlo) generations were produced and sampled at each 1000, yielding 2000 estimates with 25% discarded as burn-in. The trees were visualized and manipulated with FigTree^7^.

### Plant Materials, Mutant Identification, Overexpression Plant Production, and Growth Conditions

*Arabidopsis thaliana* ecotype Col-0 is the wild-type used in this study. The mutant T-DNA lines used herein were obtained from ABRC (*Arabidopsis* Biological Resource Center) for the *FIP* gene (Salk_080769C and Salk_069143C). To identify homozygous in the F3 generation, PCR was performed with three primers (left border 5′-AACTGCATTCCCGATCCTCT-3′; right border 5′-AAATCCTGCTCCGTCACATT-3′and LBb1.3 5′-ATTTTGCCGATTTCGGAAC-3′) using DNA samples from the leaves of three-week-old plants as template. The plants were grown in soil under control conditions (22°C, 16 h/8 h light/dark, 120 μmol m^-2^ s^-1^).

Plants overexpressing *FIP* were generated by cloning the full-length sequence of *AtFIP* (At5g02160) next to the CaMV35S promoter in the pK7WG2 Gateway vector. The sequence of *AtFIP* containing 390 bp was amplified using primers 5′-CACCATGACGATCGCACCGGCATTG-3′ and 5′-TGATTTATCAATCTGGTTAAGC-3′ and Platinum DNA Polymerase Taq High Fidelity (Thermo). The amplicon was cloned into the pENTR/D-TOPO vector (Gateway) according to the manufacturer’s instructions and subjected to DNA sequencing. The amplicon was transferred to the overexpression vector pK7WG2 using the recombination enzyme LR Clonase II (Thermo). *Agrobacterium tumefaciens* strain GV3101 was used to insert the plasmid construction into the Col-0 plants by the floral dip method (Clough and Bent, 1998). Plants overexpressing *FIP* were selected from the F3 generation in agar plates containing Murashige and Skoog (MS) medium half strength (PhytoTechnology Lab.) and 50 mg/L of kanamycin. After 2 weeks of growth on selective plates under control conditions (22°C, 16 h/8 h light/dark, 120 μmol m^-2^ s^-1^), the green seedlings were transferred to soil.

### Stress Tolerance Analysis

For light stress treatment, three-week-old plants of *FIP* overexpressing (OE) lines, *fip* knockdown mutant, and wild-type (WT) growing in soil under control conditions (22°C, 16 h/8 h light/dark, 120 μmol m^-2^ s^-1^) were transferred to high light conditions (22°C, 16 h/8 h light/dark, 400 μmol m^-2^ s^-1^) for 11 days. The plants were watered every 2 days throughout the period.

For stress assays with seedlings, seeds of OE lines, *fip* knockdown mutant, and WT were surface-sterilized, placed on 0.5X (half strength) MS agar plates (PhytoTechnology Lab.) and kept at 4°C for 2 days. The plates were then transferred to control conditions (22°C, 16 h/8 h light/dark, 120 μmol m^-2^ s^-1^) for 7 days, and then the seedlings were subjected to different stress treatments for more 10 days. The concentrations of paraquat (Methyl Viologen) for oxidative stress were 0.01, 0.05, 0.1, and 0.2 μM. The concentrations of mannitol for osmotic stress were 25, 50, 100, and 150 mM. The concentrations of NaCl for salt stress were 25, 50, 100, and 150 mM. The average root length was calculated considering the difference between the initial and final root length of all repetitions of one independent treatment. One-way ANOVA followed by Tukey’s pairwise (*P* < 0.05) was applied to show significant differences amongst all plants in different concentrations of the same treatment. Mannitol treatment with three-week-old plants was conducted using 300 mM for 24 h, and the leaves were then collected for isolation of the RNA and real-time RT-PCR analysis.

### RNA and DNA Isolation

Total leaf RNA extraction used TRIzol reagent (Invitrogen) followed by a TURBO DNase (Thermo) treatment. The first-strand cDNA was obtained using 1 μg of total RNA, ImProm-II Reverse Transcriptase (Promega), and primer oligo(dT). The DNA isolation was performed with Phenol reagent according (Edwards et al., 1991) method.

### PCR Analysis

Real-time PCR analysis was conducted using Maxima SYBR Green/ ROX qPCR Master Mix 2X (Thermo) with the Applied Biosystems StepOne real time PCR system equipment. The relative expression was calculated with the ΔCP method using *ACTIN* expression as the reference gene (Pfaffl, 2001). Significant differences between control condition and treatment were indicated by asterisks using Student’s *t*-test (*P* < 0.05). One-way ANOVA followed by Tukey’s pairwise (*P* < 0.05) was applied to show significant differences amongst all plants in different treatments. The measurements were performed using six biological replicates. The genes analyzed by PCR were as follows: *ACTIN7* (At5g09810) 5′-CTAGAGACAGCCAAGAGCAGTTC-3′ and 5′-GTTTCATGGATTCCAGGAGCTTC-3′; *FIP* (At5g02160) 5′-CATTCCCGATCCTCTTCAAA-3′ and 5′-CGAGTCCATTGCAGTTAGCA-3′; *FtsH5* (At5g42270) 5′-TTGCTGCTAGACGTGAGCTT-3′ and 5′-TGGATCATACTCCGGCATAA-3′; *D1* (AtCg00020) 5′-TCCGGAACTGCCATTCTAAC-3′ and 5′-TCCGATTCCAAAGTTCGTTC-3′; *HSP60-2* (At2g33210) 5′-CCGCATTAGTTGATGCTGCAAGTG-3′ and 5′-CGTTGGAATCTCAGTCACAACTGC-3′; *AOX1a* (At3g22370) 5′-AGCATCATGTTCCAACGACGTTTC-3′ and 5′-GCTCGACATCCATATCTCCTCTGG-3′ and *Cu-Zn-SOD* (At1g08830) 5′-GGAACTGCCACCTTCACAAT-3′ and 5′-TCCAGTAGCCAGGCTGAGTT-3′.

## Results

### *Arabidopsis* FtsH5 Protein Interacts With a Transmembrane Protein Containing a Zinc-Finger Domain

**FIGURE 1:**
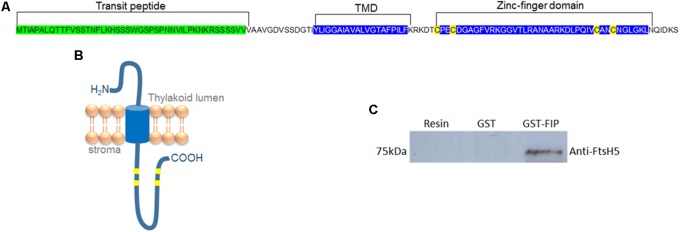
FIP is a transmembrane protein containing a zinc-finger domain and is localized in the thylakoid membrane. **(A)** Amino acid sequence of FIP protein. The transit peptide, transmembrane domain (TMD), and zinc-finger domain are marked. **(B)** Theoretical model for FIP topology predicted by PROTTER online software. FIP is anchored in thylakoids by TMD and has an amino proximal luminal domain and a carboxyl proximal stromal domain. **(C)** glutathione S-transferase (GST) pull-down assay of the FtsH5 and FIP interaction. GST and GST-FIP fusion proteins were expressed in *Escherichia coli* and purified with a GST column. GST and GST-FIP were incubated with nickel resin-purified recombinant FtsH5 fusion protein, pulled down with GST-beads, and detected using an anti-FtsH5 antibody by Western blot analysis.

The yeast two-hybrid system was employed to identify proteins involved in the stress response mechanism that potentially interacts with FtsH protein in chloroplasts, since FtsH is directly involved in stress response in plants. The complete *Arabidopsis FtsH5* gene sequence was used as bait to transform *Saccharomyces cerevisiae*. Screening against an *Arabidopsis* library resulted in the identification of 48 positive candidates that activated the histidine and adenine reporter genes (Supplementary Table S1). Among them, a hypothetical plastidial protein (AT5G02160) named FIP (FtsH5 Interacting Protein) was chosen for further characterization due to its localization and potential regulatory role provided by the zinc-finger domain. FIP possesses an N-terminal transit peptide followed by a hydrophobic domain and a zinc-finger domain (**Figures 1A,B**). Despite the presence of a zinc-finger domain (C_4_-type) with two CXXCXGXG conserved repeats, characteristic of DNAJ protein, the conserved J domain is absent in FIP. FtsH5-FIP interaction was confirmed using an *in vitro* GST pull-down assay (**Figure 1C**). The production of recombinant proteins used in this experiment is shown in Supplementary Figures S1A–D.

### FIP Is Targeted Both *in Vivo* and *in Vitro* to Chloroplasts and Inserted in Thylakoids

**FIGURE 2:**
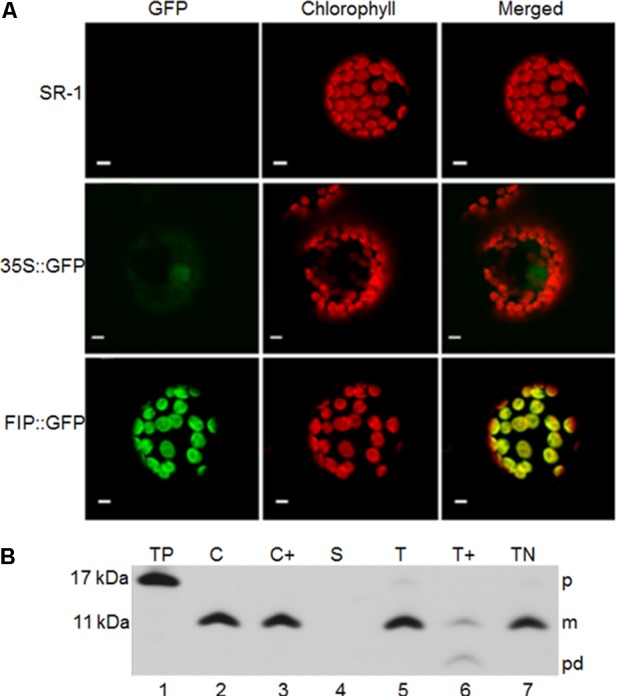
Subcellular localization of FIP. **(A)** *In vivo* chloroplast localization of the FIP::GFP fusion introduced by *Agrobacterium tumefaciens*–mediated transfection in tobacco protoplasts. Fluorescence images were obtained using a confocal laser scanning microscope. green fluorescent protein (GFP) corresponds to the fluorescence detected in the green channel. Chlorophyll, detected in the far-red channel, corresponds to the chloroplast autofluorescence signal. Chlorophyll + GFP corresponds to the merging of the far-red and green channels. Yellow represents the co-localization of green and red signals. All scale bars represent 5 μm. SR-1 corresponds to wild type *Nicotiana tabacum* non-transformed protoplasts; 35S::GFP corresponds to expression of GFP driven by the constitutive 35S promoter of Cauliflower mosaic virus (CaMV); FIP::GFP corresponds to expression of FIP::GFP fusion driven by the constitutive 35S promoter of CaMV **(B)** pFIP is imported into isolated chloroplasts and integrated into thylakoids. Radiolabeled *in vitro*-translated pFIP (TP, lane 1) was incubated with intact isolated pea chloroplasts in a reaction containing 5 mM ATP and ∼100 μE/m^-2^ sec^-1^ light at 25°C for 20 min. Intact chloroplasts were recovered from the reaction (C, lane 2) and treated with thermolysin (C+, lane 3). Untreated intact chloroplasts were fractionated into stroma (S, lane 4) and thylakoids. Thylakoid aliquots were washed with import buffer (T, lane 5), treated with thermolysin (T+, lane 6), or treated with 100 mM NaOH (TN, lane 7). Samples were analyzed by SDS–PAGE on 12.5% gels and by fluorography. The precursor, mature form, and product degradation are designated p, m, and pd, respectively, on the right side of the panels. *M*-values on the left side were estimated from their migration compared with standard marker proteins.

The subcellular localization of FIP *in vivo* was verified by fusing FIP with GFP. This construct was used to transiently transform *Nicotiana tabacum* leaves. FIP::GFP-transformed tissues displayed a GFP signal in chloroplasts (**Figure 2A**). To verify the intra-plastidial localization, the *in vitro*-translated precursor to FIP (pFIP; 13 kDa) was added to intact chloroplasts. FIP precursor was imported into the chloroplasts and processed to a 9 kDa mature form (**Figure 2B**, lane 2). The mature forms resided inside the organelle after treating recovered chloroplasts with thermolysin (**Figure 2B**, lane 3). FIP mature protein resided in the thylakoid fraction (**Figure 2B**, T, lane 5) rather than the stroma (**Figure 2B**, S, lane 4). Proper integration into the thylakoids was verified by protease treatment or extraction with 100 mM NaOH. Protease treatment produced a product of partial degradation of ∼5 kDa (**Figure 2B**, lane 6) and FIP was resistant to NaOH extraction (**Figure 2B**, TN, lane 7). These results indicated that FIP was properly integrated into thylakoid membrane.

### The FIP Zinc-Finger Domain Is Conserved and Is Only Present in Mosses and Higher Plants

**FIGURE 3:**
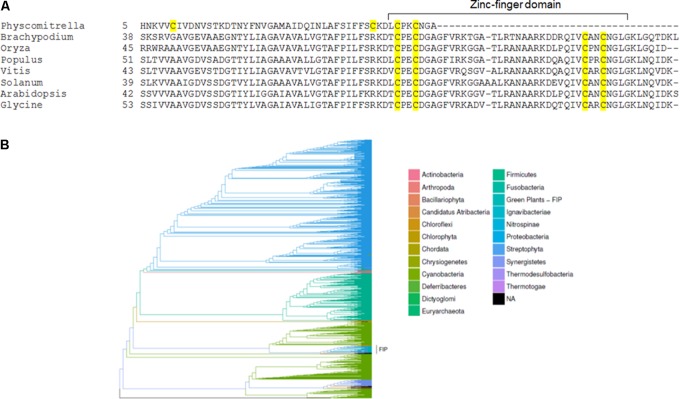
FIP homologues are restricted to terrestrial plants. **(A)** Amino acid sequence alignment of several FIP homologues. Cysteine residues are highlighted in yellow, and the zinc-finger domain is marked. Physcomitrella was included in the alignment for comparison. **(B)** Bayesian phylogenetic inference of type A FtsH homologous sequences were identified using the amino acid sequences of FtsH1 (At1g50250) and FtsH5 (At5g42270) through the blastp algorithm against the refseq_protein GenBank database, including 2073 sequences that were above the threshold *e*-value of ≤1e-180. The results produced 1 × 10^6^ MCMC (Markov chain Monte Carlo) generations sampled at each 1000 generations, yielding 1000 estimates. The cluster bearing sequences homologous to FIP was recovered as a monophyletic taxon and is highlighted.

As a first step in studying FIP evolution, we searched for FIP homologous in photosynthetic organisms and found that only mosses and higher plants carry homologous sequences. FIP amino acid alignment using higher plants sequences showed that the zinc-finger domain is conserved, in contrast to *Physcomitrella* used herein for comparisons (**Figure 3A**).

Considering FIP was found interacting with the FtsH5 protease, a phylogenetic tree for type A FtsHs was inferred and presented a monophyletic taxon composed of species possessing FIP protein, along with a few exceptions from species of the *Chlorophyta* division that lack FIP (**Figure 3B**). These findings indicated that type A FtsHs shared some features among these groups, which might have been present in FtsH proteases from the shared ancestors of green algae, mosses and higher plants before the advent of FIP.

### Knockdown Mutants and Overexpression Lines Present No Variation in *FtsH5*, *D1*, or *Actin* Transcripts and Are Phenotypically Indistinguishable From Wild-Type Under Control Conditions

**FIGURE 4:**
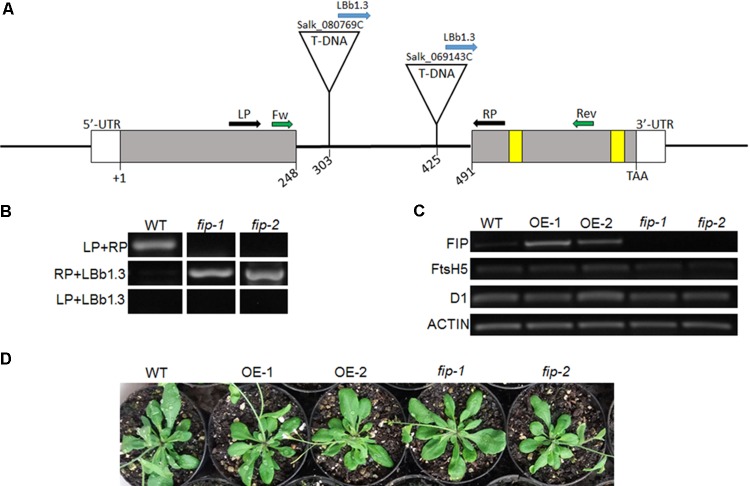
Characterization of FIP plants. **(A)** Diagram of the *FIP* gene in *Arabidopsis*. Exons are depicted as solid gray boxes. The cysteine residues of the zinc-finger domain are represented as yellow boxes. The T-DNA insertion sites in the intron region of the *FIP* gene (At5g02160) for SALK lines 080769C (*fip-1*) and 069143C (*fip-2*) are shown. The annealing sites for the primers are represented by arrows: black pair were used to confirm the mutants along with the blue; green pair were used to RT-PCR analysis. **(B)** Confirmation of mutants knockdown *FIP*. A 307 pb fragment was amplified using de LP and RP in wild-type (WT) plants. A 512 or 390 pb fragment was amplified using de RP and LBb1.3 in homozygous plants of the mutants *fip-1* and *fip-2*, respectively, and no amplification was expected using the LP and LBb1.3 primers, considering an upstream orientation of the T-DNA insertion. **(C)** Profiles of *FIP*, *FtsH5*, and *D1* mRNA accumulation in WT, OE lines, and fip knockdown mutant plants growing under control conditions for 21 days. Total RNA was isolated from leaves of *Arabidopsis thaliana*. cDNA synthesis was performed using 1 μg of total RNA. RNA accumulation was determined by semi-quantitative PCR analysis. *ACTIN* gene expression was used as a control. **(D)** Phenotypes of three-week-old WT plants, overexpressing (OE) lines, and fip knockdown mutants growing under control conditions (22°C, 16 h/8 h light/dark, 120 μmol m^-2^ s^-1^).

**FIGURE 5:**
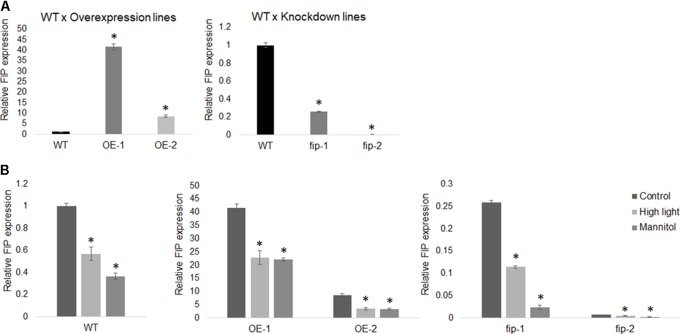
FIP expression profile. **(A)** The relative transcript levels of *FIP* in WT plants is 1.0 when compared with the overexpression lines OE-1 and OE-2 or with the knockdown mutants *fip-1* and *fip-2*. **(B)** *FIP* expression is down-regulated by high light and osmotic stresses. Three-week-old plants growing under control conditions (22°C, 16 h/8 h light/dark, 120 μmol m^-2^ s^-1^) were submitted to a 24-h stress as follows: 400 μmol m^-2^ s^-1^ (high light) or 300 mM of mannitol (osmotic stress). The relative expression was assayed by real-time RT-PCR and calculated by the ΔCP method using *ACTIN* expression as a reference gene. Values were normalized in relation to the WT expression as 1. Data are the mean ± SD (*n* = 6). Statistically significant differences are indicated by asterisks (Student’s *t*-test, *P* < 0.05) for comparisons of the OE lines and fip knockdown mutants with WT plants.

To understand the role of *FIP*, plants with altered levels of *FIP* were analyzed. Two independent *Arabidopsis* knockdown mutants for *fip* were obtained from the ABRC (Salk_080769C and Salk_069143C). Both mutants have a T-DNA insertion located in the intron of the *FIP* gene, but different positions: +303 and +425 from the ATG (+1) and before the zinc-finger domain (**Figure 4A**). The T-DNA insertion was confirmed by RT-PCR using the primers displayed in **Figures 4A,B** and Supplementary Figure S2. We designated these mutants *fip-1* and *fip-2*, respectively. Both mutants showed an extremely low level of *FIP* transcripts, as measured by RT-PCR (**Figure 4C**) and real time RT-PCR (**Figure 5A**). Two independent *Arabidopsis* transgenic OE lines *FIP* were obtained and referred to as OE-1 and OE-2. Both lines showed significantly higher transcript levels of *FIP* when compared with wild-type plants (**Figures 4C**, **5A**).

Semi-quantitative PCR analysis visually confirmed the variation in *FIP* levels observed between knockdown mutants and OE lines. However, no differences were observed between the plants when comparing *FtsH5*, *D1*, or *Actin* transcripts under unstressed control conditions (**Figure 4C**). *fip* knockdown mutants and OE lines were phenotypically indistinguishable from wild-type when grown under control conditions (22°C, 16 h/8 h light/dark, 120 μmol m^-2^ s^-1^, **Figure 4D**).

### *FIP* Expression Is Down-Regulated by Abiotic Stress

Real-time RT-PCR was performed to evaluate the expression levels of *FIP* in OE lines, *fip* knockdown mutants, and WT plants exposed to high light and osmotic stresses. For the high light stress evaluation, three-week-old plants were transferred from a control condition (22°C, 16 h/8 h light/dark, 120 μmol m^-2^ s^-1^) to a high light condition (22°C, 16 h/8 h light/dark, 400 μmol m^-2^ s^-1^) for 11 days. In addition, three-week-old plants were submitted to a 24-h stress with 300 mM of mannitol.

**FIGURE 6:**
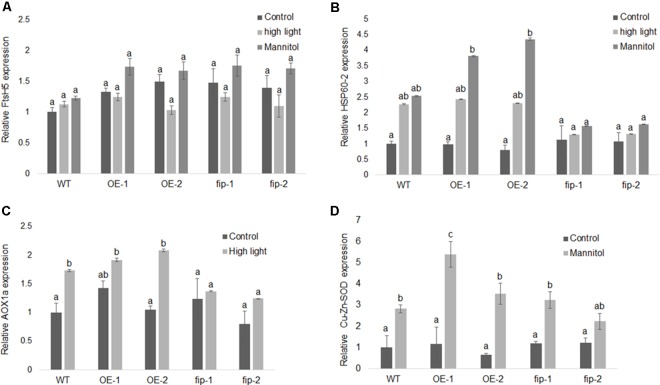
Expression of different genes in response to stress conditions. Real-time RT-PCR analysis was conducted using WT plants, *FIP* overexpression lines (OE-1 and OE-2), and *fip* knockdown mutants (*fip-1* and *fip-2*). Three-week-old plants growing under control conditions (22°C, 16 h/8 h light/dark, 120 μmol m^-2^ s^-1^) were submitted to a 24-h stress as follows: 400 μmol m^-2^ s^-1^ (high light) or 300 mM of mannitol (osmotic stress). **(A)** Relative transcript levels of *FtsH5* subjected to high light or osmotic stress. **(B)** Relative transcript levels of *HSP60-2* in plants subjected to high light or osmotic stress. **(C)** Relative transcript levels of *AOX1a* in plants subjected to high light stress. **(D)** Relative transcript levels of *Cu-Zn-SOD* in plants subjected to osmotic stress. The relative expression was calculated by the ΔCP method using *ACTIN* expression as the reference gene. Values were normalized in relation to the WT expression as 1. Data are the mean ± SD (*n* = 6). Statistically significant differences are indicated by different lower-case letters (One-way ANOVA followed by Tukey, *P* < 0.05) when comparing all plants in the same graphic.

Real-time RT-PCR results demonstrated that *FIP* was down-regulated in OE lines, *fip* knockdown mutants, and WT plants exposed to high light and osmotic stress (**Figure 5B**) when compared to control conditions. This expression pattern is consistent with the idea that FIP might have an inhibitory role in plants, since *FIP* levels are reduced during stress conditions. Considering that plants expressing different levels of FIP are not under the regulation of the same promoter (OE lines are controlled by the 35S promoter), it may indicate that FIP has some kind of post-translational regulation. The transcript levels of *FtsH5* did not show significant difference between the plants expressing different levels of *FIP* under stress conditions (**Figure 6A**). The transcript levels of the stress-responsive genes Heat Shock Protein 60 (*HSP60-2*, **Figure 6B**), Alternative Oxidase 1a (*AOX1a*, **Figure 6C**) and Cu-Zn Superoxide Dismutase (*Cu-Zn-SOD*, **Figure 6D**) were up-regulated compared with the control condition in all plants, confirming the efficiency of the stress treatments. These results suggest that *FIP* is an abiotic stress-related gene.

### Plants Expressing Low Levels of *FIP* Are More Tolerant to Abiotic Stresses

**FIGURE 7:**
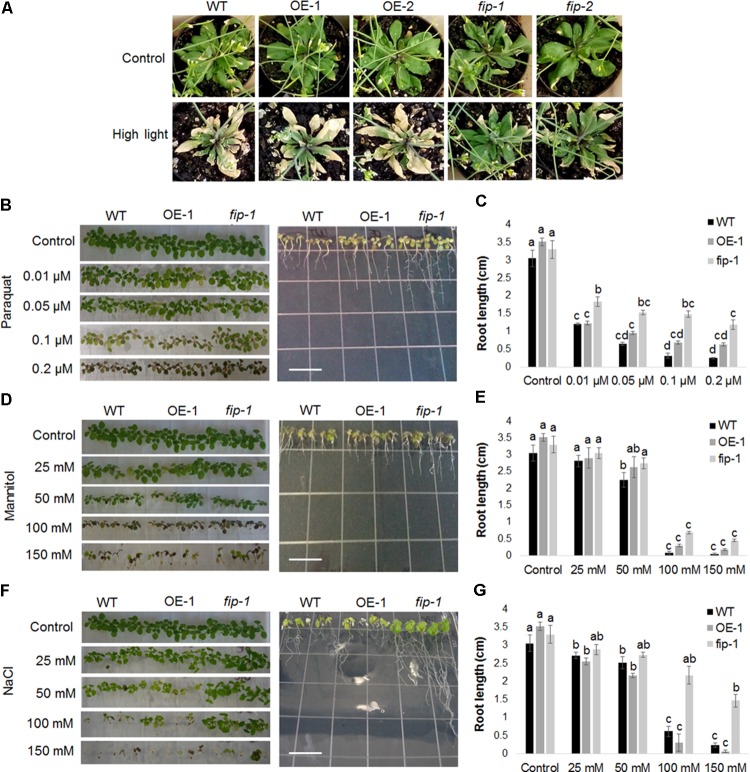
The *fip* knockdown mutants are more tolerant to abiotic stress. **(A)** Effects of high light stress on plants expressing different amounts of *FIP* transcripts. Three-week-old WT plants, *FIP* overexpression lines (OE-1 and OE-2), and fip knockdown mutants (*fip-1* and *fip-2*) growing under control conditions (22°C, 16 h/8 h light/dark, 120 μmol m^-2^ s^-1^) were subjected to high light stress (22°C, 16 h/8 h light/dark, 400 μmol m^-2^ s^-1^) for 11 days. Plants were watered every 2 days throughout the period. **(B–G)** WT plants, an *FIP* overexpression line (OE-1), and the *fip* knockdown mutant (*fip-1*) were grown in 0,5X MS plates for 7 days and then transferred to the respective stress treatment for 10 days. The plates were maintained in a room under control conditions (22°C, 16 h/8 h light/dark, 120 μmol m^-2^ s^-1^). **(B)** Plants subjected to oxidative stress. Effect of increasing amounts of paraquat (0.01, 0.05, 0.1, and 0.2 μM) on plant leaves (left). Effects of 0.2 μM of paraquat on plant root growth (right). **(C)** Average root length of plants subjected to increasing amounts of paraquat. **(D)** Plants subjected to osmotic stress. Effects of increasing amounts of mannitol (25, 50, 100, and 150 mM) on plant leaves (left). Effects of 100 mM of mannitol on plant root growth (right). **(E)** Average root length of plants subjected to increasing amounts of mannitol. **(F)** Plants subjected to salt stress. Effects of increasing amounts of NaCl (25, 50, 100, and 150 mM) on plant leaves (left). Effects of 100 mM of NaCl on plant root growth (right). **(G)** Average root length of plants subjected to increasing amounts of NaCl. All the concentrations were tested in three independent experiments with three repetitions each time with identical results. Data are the mean ± SD (*n* = 6). Statistically significant differences are indicated by different lower-case letters (One-way ANOVA followed by Tukey, *P* < 0.05). Scale bar (white) in plates means 1 cm.

Morphological and/or developmental alterations were evaluated in *fip* knockdown mutants and OE lines submitted to abiotic stress conditions. Differences in phenotype were observed in *fip* mutants submitted to a high light condition (22°C, 16 h/8 h light/dark, 400 μmol m^-2^ s^-1^ for 11 days) when compared with wild-type plants and OE lines growing under the same condition (**Figure 7A**). *fip* knockdown mutant plants became greener for a longer time, demonstrating an increased adaptation to high light conditions.

Seedlings of *FIP* OE lines, *fip* knockdown mutants, and wild-type (WT) plants were grown on agar plates containing 0.5X MS medium for 7 days and transferred to agar plates containing increasing paraquat, mannitol or NaCl concentrations for an additional 10 days. *fip* knockdown mutant seedlings growing in 0.1 μM of paraquat presented slightly improved leaf development compared with OE lines and WT plants (**Figure 7B**, left). No differences were visible between the leaves when the seedlings were grown in 0.2 μM of paraquat; however, the roots of *fip* mutants were longer and more branched than OE and WT seedlings (**Figures 7B** (right),**C**). No differences were visible between the leaves when the seedlings were grown in the presence of increasing amounts of mannitol (**Figure 7D**, left). A slightly improvement could be observed in roots length of *fip* mutants compared with OE and WT seedlings when plants were grown in 100 mM of Mannitol (**Figures 7D** (right),**E**). On the other hand, *fip* mutant seedlings leaves were clearly greener than the OE lines and WT plants in 100 mM of NaCl (**Figure 7F**, left) and the roots longer and more branched (**Figures 7F** (right),**G**). All together, these observations corroborate the low levels observed of stress-inducible genes in *fip* knockdown mutants when compared to OE lines and WT plants (**Figures 6B–D**), clearly demonstrating that FIP is involved in the abiotic stresses response.

## Discussion

FtsH proteins are directly involved in stress responses. In microorganisms, FtsHs, under abiotic stress conditions, are involved in protein quality control. *Lactobacillus plantarum* FtsH mutants exhibit sensitivity to high temperature and increased salt concentrations (Bove et al., 2012). *Synechocystis* cells lacking a homologue of FtsH show growth inhibition under heat and light stress (Kamata et al., 2005). In *Arabidopsis*, thylakoid FtsH activity is directly correlated with light stress and photosystem II quality control (Khatoon et al., 2009; Kirchhoff et al., 2011; Yoshioka and Yamamoto, 2011; Herbstova et al., 2012). The use of FtsH5 protease as bait into the yeast two-hybrid system was presented as a successful strategy for the discovery of protein factors involved in the stress response. FIP (At5g02160) was found to interact with FtsH5 in our two-hybrid system approach and its amino acid sequence can be divided into three distinct regions: an amino proximal cleavable transit peptide, a hydrophobic membrane anchoring region, and a carboxyl proximal zinc-finger domain (**Figures 1A,B**). FIP and FtsH5 are both inserted in the thylakoid membrane. *In vivo* experiments using FIP fused to the green fluorescent protein (GFP) allowed us to clearly observe the plastid localization (**Figure 2A**). The *in vitro* import into isolated chloroplasts and subsequent separation of the membranous fraction resulted in FIP insertion in the thylakoid membrane (**Figure 2B**), in agreement with a proteolytic study in thylakoids (Peltier et al., 2004).

Based on our observation that FIP homologues are only present in mosses and higher plants (**Figure 3B**), it is possible to speculate that FIP might have evolved as an adaptation to the terrestrial environment. In addition, FIP would have provided a mechanism for fine tuning in plants in the context of these new stresses, allowing more precise and dynamic control of the stress response mechanism. Moreover, the sequence conservation in the zinc-finger domain amongst the FIP carrying species (**Figure 1B**) reinforcing the importance of their protein-protein interaction ability, and the possibility of FIP to present a regulatory function in plants.

The analysis of plants expressing different levels of *FIP* demonstrated that *fip* knockdown mutants exhibited enhanced phenotypic tolerance to abiotic stress conditions (**Figures 7A–G**) and that the transcript levels of *FIP* were down-regulated under all stress conditions for all plants (OE lines, *fip* knockdown mutants, and WT plants, **Figure 5B**). This profile is consistent with previous results available in the public data (Supplementary Figures S3A–D). As expected, stress-responsive genes were up-regulated in all treatments when compared to the control (**Figures 6B–D**), which is consistent with their protective role to prevent damage (Moller, 2001; Bender et al., 2011; Vanlerberghe, 2013). However, the transcript levels of these genes were slightly reduced in *fip* knockdown mutants when compared with OE lines and WT plants (**Figures 6B–D**), corroborating the observations that *fip* knockdown mutants are more tolerant to abiotic stresses.

In chloroplasts, protease activity is continuously regulated by chaperones that act coordinately to assure the protein quality control system (Nishimura et al., 2017), which is essential for plant development (Lu, 2016). FIP possess a zinc-finger domain (type C_4_) with two CXXCXGXG conserved repeats (**Figure 1A**), a similar structure found in DNAJ proteins. However, FIP lacks the conserved J domain. In DNAJ proteins, the zinc finger interacts with the substrate while J domain interacts with Hsp70 (Kampinga and Craig, 2010; Veyel et al., 2014; Wang et al., 2015). In the absence of a conserved J domain, as observed in FIP, DNAJ-like proteins usually show a chaperone-like role without Hsp70, by substrate interaction and stabilization. For example, the *Orange* gene codes the plastid-localized protein OR that carries a cysteine-rich zinc finger motif, as observed in DNAJ proteins, but not the J domain (Lu et al., 2006). OR is involved in the regulation of phytoene synthase in controlling carotenoid biosynthesis (Zhou et al., 2015). Also, CYO1 protein has a zinc finger domain (type C_4_) similar to the domain found in DNAJ of *E. coli* (Shimada et al., 2007) and is related to thylakoid formation and interacts with the LHCB1 protein (Zagari et al., 2017). In another study, (Hartings et al., 2017) demonstrate that HCF222, a DNAJ-like protein, is related to thylakoid formation.

The involvement of FIP in abiotic stress response is clearly observed in our results, in agreement with previous studies with DNAJ and zinc finger proteins (Chen et al., 2010; Kong et al., 2014; Wang et al., 2014, 2015, 2016; Xia et al., 2014). Despite the co-localization and observation of interaction in the yeast two-hybrid system, it is still too early to directly relate the increase in abiotic stress responses, due to reduced FIP levels, to FtsH activity in chloroplasts. However, models for protease control, described in the literature (Nishimura et al., 2017) strong suggest that FtsH protease activity control can count on additional regulators since, so far, no regulation mechanism for FtsH protease in plants has been described. Additional studies to investigate the precise role o FIP interaction with FtsH5 are underway.

## Author Contributions

KL, RR, MS, WB, and MS-F conceived the topic. KL, RR and MS-F wrote the manuscript.

## Conflict of Interest Statement

The authors declare that the research was conducted in the absence of any commercial or financial relationships that could be construed as a potential conflict of interest.

## Footnotes

1 http://www.arabidopsis.org

2 http://www.ch.embnet.org/software/TMPRED_form.html

3 https://www.arabidopsis.org/portals/expression/microarray/ATGenExpress.jsp

4 http://www.ncbi.nlm.nih.gov/genbank

5 http://www.phytozome.net/

6 http://translatorx.co.uk/

7 http://tree.bio.ed.ac.uk/software/figtree/
