# Supplementary material for: The Zinc-Finger Thylakoid-Membrane Protein FIP Is Involved With Abiotic Stress Response in Arabidopsis thaliana
Source: Front Plant Sci. 2018 Apr 18;9:504. doi: 10.3389/fpls.2018.00504 (PMC5915565; doi:10.3389/fpls.2018.00504)
Supplement: Supplementary file 1 [file Table_1.DOCX]

**Supplemental Material**

Supplemental Figure 1


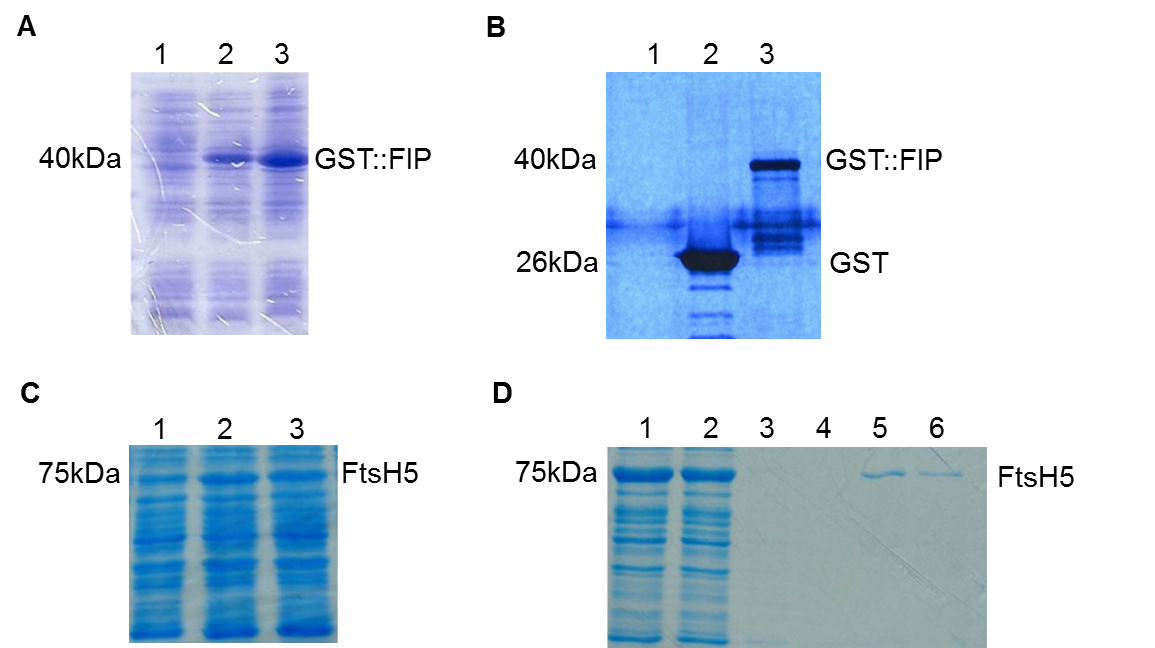


**Figure S1.** Protein production for GST-Pull Down experiments. (A) GST::FIP production where, 1) not induced, 2) 2h of induction, 3) 4h of induction. (B) GST::FIP confirmation where, 1) none, 2) GST only, 3) GST::FIP. (C) HIS::FtsH5 production where, 1) not induced, 2) 2h of induction, 3) 4h of induction. (D) HIS::FtsH5 purification where, 1) pellet, 2) soluble, 3-4) washes, 5-6) elution. The proteins were produced in E. coli strain BL21 (DE) Rosetta and the induction performed adding [Isopropyl β-D-1-thiogalactopyranoside](https://en.wikipedia.org/wiki/Isopropyl_%CE%B2-D-1-thiogalactopyranoside) (IPTG) to medium. The gels were stained with Comassie blue (A, C and D) or Silver (B).

Supplemental Figure 2


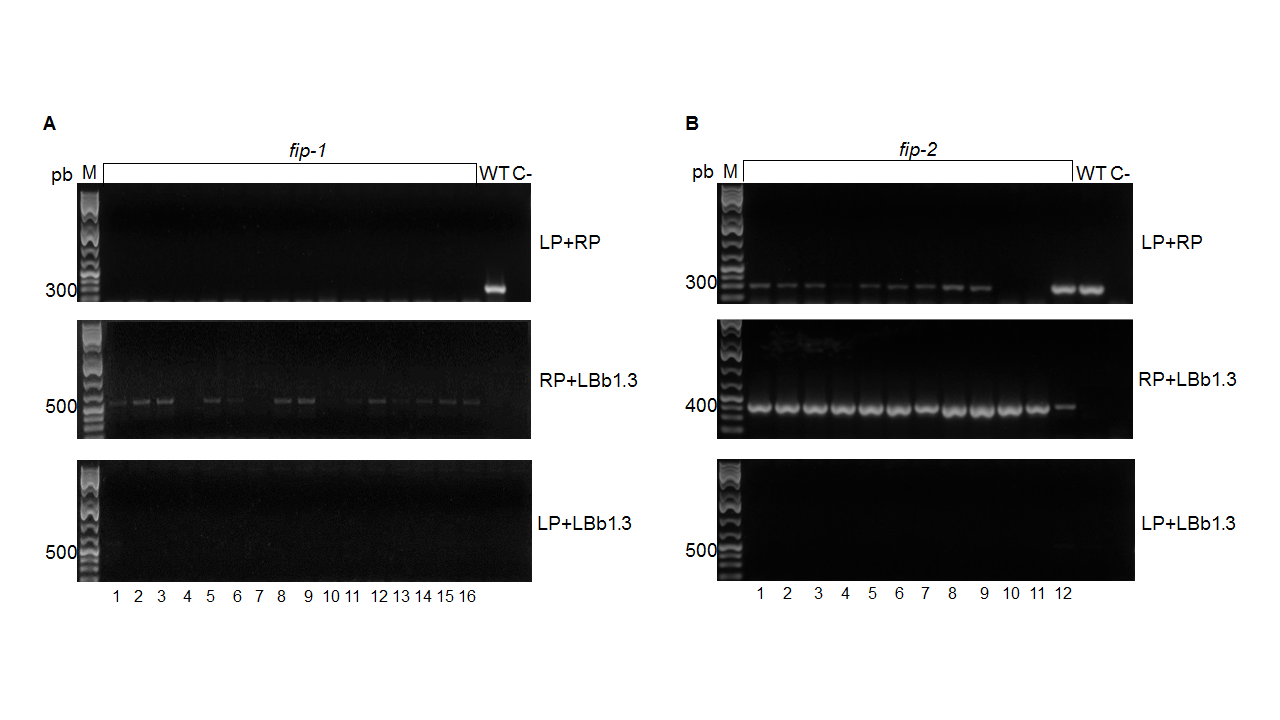


**Figure S2.** Confirmation of mutants knockdown *FIP*. Amplification of different fragment sizes using different combinations of primers and RT-PCR. The DNA was isolated from three-week-old plants leaves of mutants knockdown *FIP*. The primers used were LBb1.3 that amplifies a 250 pb fragment of the T-DNA; and LP (left) and RP (right) primers flanking the T-DNA insertion region of both mutants according the diagram in Figure 4A. A 307 pb fragment was expected to amplify using de LP and RP in WT and heterozygous plants. A 512 or 390 pb fragment was expected to amplify using de RP and LBb1.3 in homo and heterozygous plants of the mutants *fip-1* and *fip-2*, respectively, and no amplification was expected using the LP and LBb1.3 primers, considering an upstream orientation of the T-DNA insertion. (A) Amplifications obtained from DNA of *fip-1* (Salk_080769). Lanes 1-16 are independent individuals tested from T3 generation. (B) Amplifications obtained from DNA of *fip-2* (Salk_069143). Lanes 1-12 are independent individuals tested from T3 generation. WT means DNA sample from wild-type plants. C- means the same RT-PCR reaction without any source of DNA. Fragments were separated by electrophoresis in 1% agarose gels.

Supplemental Figure 3


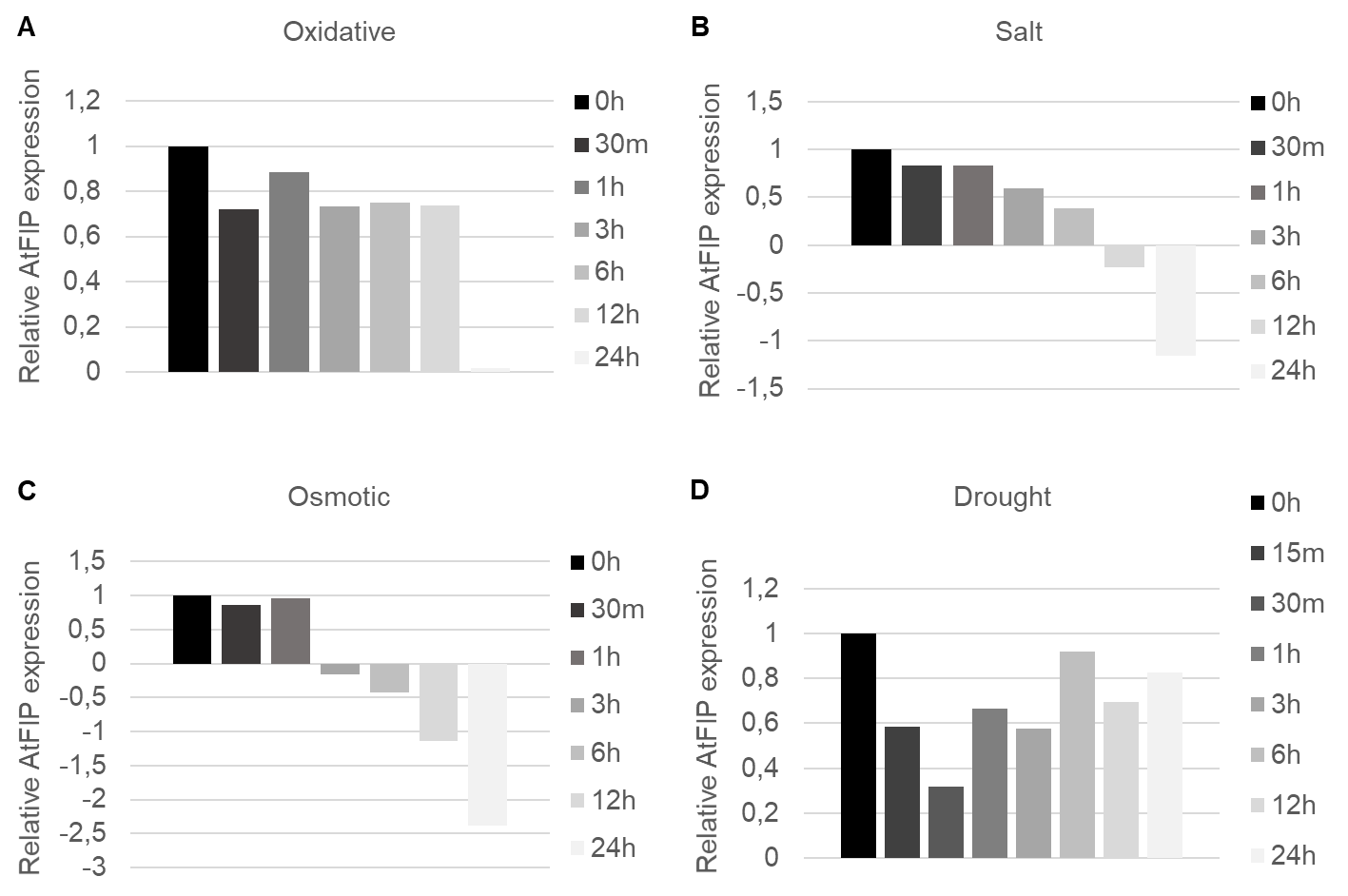


**Figure S3.** FIP expression profile in wild-type plants under abiotic stress. The analyzed data comes from the *AtGenExpress* Project, deposited in the TAIR database (*The Arabidopsis Information Resourse* – www.arabidopsis.org). Relative AtFIP expression is the contrast between control and treatment sets of wild-type plants, for each stress condition, in terms of logarithmic fold change (log2fc). Legend represents the time series, which ranges from 0 to 24 hours. (A) Oxidative stress with 10µM Methyl viologen (B) Salt stress with 150mM NaCl. (C) Osmotic stress with 300mM Mannitol. (D) Drought stress until plants lose 10% of their fresh weight.

**Supplemental Table 1**

**Table S1:** Blast results using DNA sequence from colonies obtained in yeast two-hybrid experiment using Arabidopsis thaliana FtsH5 as a bait

| **Name** | **Function** | **Localization (predicted)** |
| --- | --- | --- |
| ATSERPIN1 | Protease inhibitor | AP |
| Alfa-amylase 3 | Amylase | CL |
| Beta fructofuranosidase | Invertase | CL |
| Metallothionein | Heavy metal binding | CL |
| Ornithine carbamoyltransferase | Citrulin synthesis | CL |
| **Unknown protein (FIP)** | **-** | **CL** |
| Ribulose-bifosfato carboxilase | Photosynthesis | CL |
| Subunidade K do fotossistema I | Photosynthesis | CL |
| TOC64 | Protein translocation | CL |
| Cafeoil-CoA 3-O-metiltransferase | Lignin formation | CT |
| Thioredoxin H type 3 | Disulphide bridges reduction | CT |
| LPT protein family | Protease inhibitor | EX |
| SNARE | Vesicle transport | GC |
| Atpip1 | Aquaporin | MB |
| 3-Cetoacil-coa thiolase | Glycolysis | MT |
| Unknown protein | - | MT |
| Disease resistance protein | Defense | NC |
| Oxoglutarate dehydrogenase | Citric acid cycle | ND |
| RGLG1 (Ring Domain Ligase1); | Zinc binding | ND |
| DIS3 | Cytoskeleton | NL |
| Unknown protein | - | NL |
| RTV1 (Related To Vernalization1) | Transcription factor | NL |
| Glyceraldehyde-3-phosphate dehydrogenase | Glycolytic pathway | PX |

Note – Localization column indicates the subcellular prediction using WolfPSORT. AP: Apoplast; CL: Chloroplast; CT: Cytoplasm; EX: Extracellular; GC: Golgi complex; MB: Plasma membrane; MT: Mitochondrion; ND: Not determined; NL: Nucleus; PX: Peroxisome.
